# Neural networks for genetic epidemiology: past, present, and future

**DOI:** 10.1186/1756-0381-1-3

**Published:** 2008-07-17

**Authors:** Alison A Motsinger-Reif, Marylyn D Ritchie

**Affiliations:** 1 Bioinformatics Research Center, Department of Statistics, North Carolina State University, Raleigh, NC, USA; 2 Center for Human Genetics Research, Department of Molecular Physiology and Biophysics, Vanderbilt University, Nashville, TN, USA

## Abstract

During the past two decades, the field of human genetics has experienced an information explosion. The completion of the human genome project and the development of high throughput SNP technologies have created a wealth of data; however, the analysis and interpretation of these data have created a research bottleneck. While technology facilitates the measurement of hundreds or thousands of genes, statistical and computational methodologies are lacking for the analysis of these data. New statistical methods and variable selection strategies must be explored for identifying disease susceptibility genes for common, complex diseases. Neural networks (NN) are a class of pattern recognition methods that have been successfully implemented for data mining and prediction in a variety of fields. The application of NN for statistical genetics studies is an active area of research. Neural networks have been applied in both linkage and association analysis for the identification of disease susceptibility genes.

In the current review, we consider how NN have been used for both linkage and association analyses in genetic epidemiology. We discuss both the successes of these initial NN applications, and the questions that arose during the previous studies. Finally, we introduce evolutionary computing strategies, Genetic Programming Neural Networks (GPNN) and Grammatical Evolution Neural Networks (GENN), for using NN in association studies of complex human diseases that address some of the caveats illuminated by previous work.

## Introduction

The identification of disease susceptibility genes for complex, multifactorial disease is arguably the most difficult challenge facing human geneticists today [1]. Most common diseases are the result of complex interactions among multiple genetic factors in addition to a collection of environmental exposures [2]. This has been documented by Ming and Muenke who compiled a list of diseases with known epistatic interactions [3]. Traditional gene mapping studies utilize one of two possible research strategies: linkage or association. Linkage analysis determines whether a chromosomal region is preferentially inherited by offspring with the trait of interest by using genotype and phenotype data from multiple biologically-related family members. Linkage analysis capitalizes on the fact that, as a causative gene(s) segregates through a family kindred, other markers nearby on the same chromosome tend to segregate together (are in linkage) with the causative gene due to the lack of recombination in that region. Association analysis, on the other hand, describes the use of case-control, cohort, or even family data to statistically relate genetic variations to a disease/phenotype. While each of these approaches has been very effective in identifying disease genes in rare, Mendelian disorders, there are additional challenges when studying common, complex diseases. To aid readers less familiar with the terminology used in genetic epidemiology, Table 1 provides a glossary of terms used in the current review.

**Table 1:** Glossary of Statistical Genetics Terms

**Term**	**Definition**
Allele	One member of a series of different forms of a gene
Association study	The use of case-control, cohort, or even family data to statistically relate genetic variations to a disease/phenotype
Chromosome	A singular, physical piece of DNA, which can contain many genes and regulatory elements
Epistasis	Gene-gene interaction; as a deviation from additivity in the effect of alleles at different loci with respect to their contribution to a phenotype
Gene	A heritable unit; a region of genomic sequence which is associated with regulatory, transcribed, and/or other functional regions
Genotype	Specific allele combinations for an individual
Genotyping	The experimental determination of sequence variations
Linkage study	The use of genotype and phenotype information from multiple biologically-related family members to determine whether a chromosomal region is preferentially inherited by offspring with the trait of interest
Locus	A fixed position on a chromosome
Mendelian disease	A genetic disease that is caused by a single locus, and displays a pattern of inheritance in line with Mendel's Laws
Phenotype	A measurable trait for an individual
Pedigree	Multiple biologically-related individuals with known familial relationships
Single Nucleotide Polymorphism (SNP)	A DNA sequence variation; the smallest unit of variation in the genome

Complex genetic diseases present several difficult challenges for linkage analysis. First, there is no clear mode of inheritance for most of these diseases. Many linkage methods require the specification of a genetic model (mode of inheritance) for the analysis. Model-independent methods have been developed to account for this, but suffer a reduction in power compared to parametric counterparts. Second, it is likely that multiple loci with varying effects interact to yield an increased risk of disease. The degree and type of interactions will influence the ability to detect genes through linkage analysis. If the interacting loci exhibit strong independent effects, they should be detectable by linkage analysis. However, linkage analysis may not be able to detect a locus that has a small effect [4]. It is hypothesized that interactions between disease susceptibility genes with minimal main effects will be the norm rather than the exception for many common diseases [2,5-7]. Thus, linkage methods alone may not be able to detect disease susceptibility genes for common, complex diseases.

Similarly, potential caveats exist for association analysis methods for detecting interactions. First, with methods such as logistic regression, the interaction effects must be explicitly modeled. That is, one needs to have knowledge about the interaction that is being tested in advance. Second, current association analysis methods were developed to detect single-locus main effects and thus were not designed for detecting complex gene-gene interactions or epistasis [7]. Epistasis has been discussed in the literature for many years [8,9] and, when properly investigated, is often identified in genetic association studies [7]. Third, even if the statistical methodology can detect and model the interaction effects in addition to main effects, the selection of variables to evaluate is a major computational challenge. As genomic technologies advance and high-throughput genotyping becomes increasingly more affordable, the dimensionality involved in the evaluation of combinations of many such variables quickly diminishes the usefulness of traditional, parametric statistical methods. As the number of genetic or environmental factors increases and the number of possible interactions increases exponentially, many contingency table cells will be left with very few, if any, data points. This is known as the curse of dimensionality [10]. In logistic regression analysis, this can result in increased type I errors and parameter estimates with very large standard errors [11]. Traditional approaches are limited in their ability to deal with many factors and simultaneously fail to characterize epistasis models in the absence of main effects due to the hierarchical model building process [6]. This results in an increase in type II errors (false negatives) and decreased power [12], especially in relatively small datasets. For many association studies, it would be infeasible to analyze each SNP separately as well as all possible combinations of SNPs for many association studies. Therefore, careful selection of the best SNPs to evaluate must be performed prior to statistical testing.

To deal with the limitations of traditional linkage and association analysis methods in detecting susceptibility genes, alternative statistical and computational approaches must be explored. In the search for new statistical methodologies, it is helpful to look to other fields that deal with similar problems in modeling data with many variables and complex interactions. Certainly some of the approaches that have been successful in other areas such as computer science, economics, and engineering, may translate to genetic epidemiology.

### Neural Networks

A computational approach that has been proposed for the study of disease susceptibility genes is neural networks (NN). NN are a class of pattern recognition methods developed in the 1940's to model the neuron, the basic functional unit of the brain [13]. The motivation behind the continued development of NN is driven by problems that conventional computers cannot solve, where the human brain is quite capable. This is due to the architectural differences between the human brain, which functions in parallel, and the computer, which traditionally considers data sequentially. Therefore, NN are used to construct a collection of simple analog processors in parallel to take an input pattern and generate an output signal [14]. The brain and NN share several properties. Both have processing elements that are referred to as neurons. The connections between neurons occur at synapses with varying strengths. It is this strength that is associated with learning. Finally, excitatory and inhibitory potentials can be conducted by neurons in the brain and in most NN [15].

**Figure 1:**
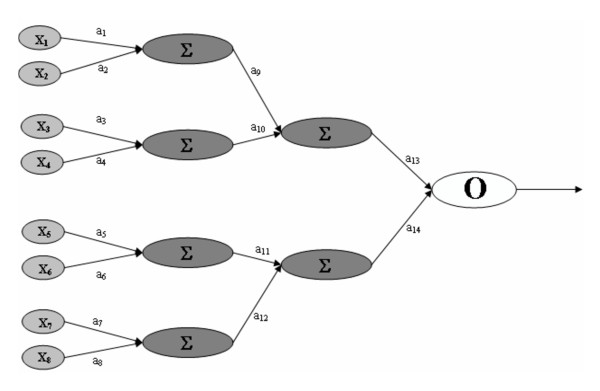
**A Typical Feed-Forward NN**. A feed-forward neural network with one input layer consisting of eight nodes (X_i_), two hidden layers with four and two nodes respectively (Σ), and one output layer (O). The connections between layers have associated connection strengths or weights (a_i_).

Neural networks can be thought of as an acyclic directed graph (Figure 1). They consist of nodes that represent the processing elements (or neurons), arcs that represent the connections of the nodes (or synaptic connections), and directionality on the arcs that represent the flow of information [14]. The processing elements, or nodes, are arranged in layers. The diagram in Figure 1 consists of four layers: an input layer, two hidden layers, and an output layer. The input layer receives the external pattern vector that is to be processed by the network. Each node (X_i_) in the input layer is then connected to one or more nodes in a hidden layer (Σ). The nodes in the hidden layer are in turn connected to nodes in additional hidden layers or to each output node (O). The number of hidden layers can range from zero to as many as computationally feasible. In Figure 1, there are four nodes in the first hidden layer and two nodes in the second hidden layer. Each network connection has a weight (a_i_) or coefficient associated with it. The signal is conducted from the input layer through the hidden layers to the output layer. The output layer, which often consists of a single node, generates an output signal that is then used to classify the input pattern.

The input pattern vector that is propagated through the network can consist of continuous input values or discrete input values. The output node(s) can also be continuous or discrete values. Designing the network architecture must take into account the representation of the input pattern vector and how it will interact with the network while propagating information through the network [14]. Thus, the data representation scheme must be suitable to detect the features of the input pattern vector such that it produces the correct output signal. A large field of neural network design has been devoted to the question of proper data representation. More detail regarding the caveats and considerations in this task can be found in Skapura [14].

As mentioned earlier, learning and memory are thought to be associated with the strength of the synapse. In NN the connections (or synaptic weights) are representative of the strength of the synapse. Therefore, setting the connection strengths is what allows the network to learn [16]. The connection strengths, together with their inputs lead to an activity level. This activity is then used as input for the next layer of the NN [17]. NN often function with back propagation types of error minimization functions, also called gradient descent. Since learning is associated with the synaptic weights, back propagation algorithms minimize the error by changing the weights following each pass through the network. This "hill-climbing" algorithm makes small changes to the weights until it reaches a value to which any change makes the error higher, indicating that the error has been minimized [14].

Several research groups have suggested NN as a useful approach for genetic epidemiology. The features of NN that make them appealing are: 1) they are able to handle large quantities of data, 2) they are universal function approximators and therefore should be able to approximate any genetic penetrance function, 3) they are genetic model free, therefore no assumptions of the genetic model need to be made, 4) they can be implemented in a variety of software packages. Therefore, using NN does not necessarily require a computer programmer and the development of new statistical software. Through the remainder of this review, we briefly discuss several studies in genetic epidemiology where NN have been applied. We look at the types of studies where NN were implemented, the different data types analyzed, and we give an overview of results from the different studies. Next, we compile the results to demonstrate that, while NN appear to be a promising approach for genetic epidemiology, questions remain that need to be addressed. Finally, we introduce evolutionary computation strategies that will improve the utility of NN for genetic epidemiology studies by addressing the previous limitations.

## Neural Networks in genetic epidemiology

As mentioned earlier, there are two main analytical approached in human genetics for the identification of disease susceptibility genes: linkage analysis and association analysis. NN have been utilized for both types of analyses. The design of the NN architecture varies depending on whether the focus is detecting linkage between a marker and a disease locus (as in linkage analysis), or detecting linkage disequilibrium between a marker and a disease locus (as in association analysis). Thus, while NN can be used for both types of studies, the inputs and outputs of the NN will be dependent upon the type of study that is conducted. Presently, we examine NN approaches in linkage analysis followed by a review of NN for association analysis. The studies reviewed are summarized in Table 2.

**Table 2:** Summary of NN Studies Reviewed

Publication	Input		Output		Hidden Layer	
	
	Type	Coding	Type	Coding	Number Layers	Number Nodes
Bhat et al. 1999	Binary	0 = absence of allele	Binary	1/0/0 = unaffected	1	15
		1 = presence of allele		0/1/0 = mildly affected		
				0/0/1 = severely affected		
Bush et al 2005	Discrete	-1, -1 = 1/1 genotype	Binary	0 = unaffected	GP evolved	
		0, + 2 = 1/2 genotype		1 = affected		
		+1, -1 = 2/2 genotype				
Costello et al. 2003	Dicrete	Varied	Binary	0 = unaffected	Multiple variations	
				1 = affected		
Curtis et al. 2001	Discrete	0 = AA genotype	Binary	0 = unaffected	2	3
		1 = AB genotype		1 = affected		
		2 = BB genotype				
Curtis 2007	Discrete	0 = AA genotype	Binary	0 = unaffected	2	3
		1 = AB genotype		1 = affected		
		2 = BB genotype				
Giachino et al 2007	Discrete and Continuous	Categorical values of genotypes and clinical features	Binary	0 = unaffected	1	unknown
				1 = affected		
Li et al. 1999	Discrete	IBD sharing	Binary	0/1 = concordant or not	Multiple variations	
		+1= shared allele		0/1 = affected or unaffected		
		-1 = unshared allele				
		0 = uninformative				
Lin et al 2006	Discrete	Categories of genotype combinations	Binary	0 = non-response	Multiple variations	
				1 = response		
Lucek and Ott 1997	Binary	0 = absence of allele	Binary	4 nodes for each trait (20 total nodes)	1	70
		1 = presence of allele		0 = quantitative trait off		
				1 = quantitative trait on		
Lucek et al. 1998	Discrete	IBD sharing	Binary	+1,+1 = target output	1	√220
		+1= shared allele		0, +1 = noise		
		-1 = unshared allele				
		0 = uninformative				
Marinov and Weeks 2001	Discrete	IBD sharing	Binary	+1,+1 = target output	1	√220
		+1= shared allele		0, +1 = noise		
		-1 = unshared allele				
		0 = uninformative				
Matchenko-Shimko and Dube 2006	Discrete	Three combinations of possible allele combinations, transformed to a 0–1 range	Binary	0 = control	Multiple variations	
				1 = case		
Motsinger et al (2006a)	Discrete	-1, -1 = 1/1 genotype	Binary	0 = unaffected	GP Evolved	
		0, + 2 = 1/2 genotype		1 = affected		
		+1, -1 = 2/2 genotype				
Motsinger et al (2006b)	Discrete	-1, -1 = 1/1 genotype	Binary	0 = unaffected	GE Evolved	
		0, + 2 = 1/2 genotype		1 = affected		
		+1, -1 = 2/2 genotype				
North et al 2003	Discrete	0 = AA genotype	Binary	0 = unaffected	Multiple Variations	
		1 = AB genotype		1 = affected		
		2 = BB genotype				
Ott 2001	Discrete	-1, -1 = 1/1 genotype	Binary	0 = unaffected	NA	
		0, +2 = 1/2 genotype		1 = affected		
		+1, -1 = 2/2 genotype				
Pankratz et al. 2001	Discrete	IBD sharing	Binary	1/1 = affected/affected	1	4
		+1 = shared allele		0/1 = affected/unaffected		
		-1 = unshared allele				
		0 = uninformative				
Penco et al 2005	Discrete	Categories of allele combinations at each genotype	Binary	0 = unaffected	Multiple variations, including and evolutionary process	
				1 = affected		
Pociot et al. 2004	Discrete	Number of categories per sliding window	Binary	0 = unaffected	Multiple variations	
				1 = affected		
Ritchie et al. 2003	Discrete	-1, -1 = 1/1 genotype	Binary	0 = unaffected	GP evolved	
		0, + 2 = 1/2 genotype		1 = affected		
		+1, -1 = 2/2 genotype				
Saccone et al. 1999	Discrete	IBD sharing	Binary	1/1 = affected/affected	18 variations	
		+1= shared allele		0/1 = affected/unaffected		
		-1 = unshared allele				
		0 = uninformative				
Serretti and Smeraldi 2004	Discrete	SERPR*l/l = 1	Binary	0 = nonresponse	1	7
		SERPR*l/s = 2		1 = response		
		SERPR*s/s = 2				
		TPH*C/C = 1				
		TPH*C/A = 2				
		TPH*A/A = 2				
Shoemaker et al. 2001	Varied	Varied	Binary	0 = unaffected	1	unknown
				1 = affected		
Tomita et al 2004	Discrete	Homozygous for major allele = (0.1, 0.1)	Binary	0.9 = case	Multiple variations	
		Heterozygous = (0.1, 0.9)		0.1 = control		
		Homozygous for minor allele = (0.9, 0.9)				
Zandi et al. 2001	Contin.	Pedigree-specific NPL scores	Binary	1,1 = case pedigree	15 variations	
				1,0 = control pedigree		

### Neural Networks for linkage analysis

Several research groups have explored NN as an analysis tool for linkage studies [4,18-25]. NN have not been widely accepted by the field as a valid approach for linkage analysis. One reason for this may be due to a fundamental flaw in logic. NN are primarily designed for classification tasks, where linkage analysis is hypothesis testing that a certain gene region contains a disease susceptibility gene. Also NN are often viewed as a "black box" whereby one cannot easily interpret the model and the influences of the input variables. These challenges have been dealt with in the previous applications of NN using techniques such as calculating a contribution value to measure the linkage signal for each of the susceptibility loci [20]. Alternatively, NN may be applicable to linkage analysis under certain conditions, and the lack of widespread adoption of the technique may be due to the high degree of variability of success in previous NN applications for linkage analysis. While the raw data are similar among all linkage studies analyzed by NN, many other aspects of the analyses are quite different. For example, the primary questions and hypotheses, the encoding of the data, as well as the architecture of the NN were different in most of the studies reviewed.

Although each study that utilized NN for linkage analysis had the same underlying goal, the primary questions and hypotheses were different for each paper. Lucek and Ott [4] planned to use NN to identify genes involved in interactions with other loci. Their hypothesis was that these interacting loci would be distinguishable from loci with no effects. Several groups attempted to use NN to identify sets of markers involved in complex disease etiology [18-21,23,25]. For others, the goal was to map inputs to the classification of a sibling pair as either discordant or concordant based on allele sharing information and environmental risk factors [22,24].

For a typical linkage analysis, the raw data consist of genotypes at many genetic markers for a collection of individuals from one or more families and a measured phenotype that is either discrete or continuous. In terms of NN architecture, the genotypes are used as NN input, and the phenotype values are used as NN output. There are a number of encoding strategies that have been employed for both inputs and outputs of a NN for linkage analysis. One type of encoding for NN inputs is based on the presence or absence of a marker allele [4,18,23]. The data are coded 1 = allele present, 0 = allele absent for each marker in the data set. Another encoding scheme that has been used involves representing the data according to identity-by-descent (IBD) sharing, such that x = 1 for sharing an allele, x = -1 for not sharing the allele, and x = 0 for uninformative. This coding scheme has been the more common type of encoding for linkage analysis with NN [19-22,24]. Finally, NPL scores (a measure of allele sharing used in non-parametric linkage analysis) could also be used as inputs (predictors) of the NN [25].

The encoding of NN outputs in linkage analysis has also been quite variable. When the target output of the NN is disease status, a common type of encoding is a simple binary output unit, using the value 1 = affected and 0 = unaffected [18,23]. An alternative to this approach is to use two output nodes such that one node is for signal and the other node is for noise. For data where you expect to detect a signal, the output pattern would be (+1, +1). In data where there is only noise, the output pattern would be (0, +1) [20,21]. A different use of two output nodes can be employed where one node represents sib pair concordance and the other represents affected status [19]. Another alternative for two output nodes involves using the classification of sib pairs as output to the NN. Here, instead of affected and unaffected individuals, the goal is the classification of the sib pair as either "affected-affected" (concordant sib pair) or "affected-unaffected" (discordant sib pair) [22,24]. Another variation on two output nodes is 1,1 for case pedigrees and 1,0 for control pedigrees [25].

For quantitative trait output values, one encoding scheme utilizes one output unit for each quartile of the trait. Then each quartile is coded as either "on" (x = 1) if the trait falls in that quartile, or "off" (x = 0) otherwise [4]. Another way to encode output data is a coding scheme based on the degree of the phenotype. For example, an output layer can be designed with three nodes, each representing one of three phenotypes: unaffected, mildly affected, or severely affected [18]. Since most studies reviewed used a different input and/or output-encoding scheme, it is not clear at this point that there is an optimal way for encoding linkage data for a NN analysis. The type of encoding chosen will affect the interpretation of the results. Thus, for different questions, different encoding strategies will be optimal.

Another important aspect of NN analysis is the design of NN architecture. There is no rule of thumb for how to select the best NN architecture. Several different strategies have been used in genetic epidemiology. One approach is to select a single NN architecture for the NN analysis. This architecture will consist of an input layer (with a number of input nodes based on the input encoding scheme selected), one or more hidden layers with an arbitrary number of hidden units and an output layer (with the number of nodes being determined by the strategy previously selected to represent the data). Lucek and Ott [4], for example, used a NN architecture consisting of 367 input nodes, 70 hidden nodes in one hidden layer, and 20 output nodes (five quantitative traits and four nodes per trait to represent quartiles). Another variation on this approach includes one input layer, one hidden layer with the number of hidden nodes specified by the square root of the number of inputs, and one output layer. This approach was used by Lucek et al. [20] who used 220 input nodes, √220 hidden nodes in one hidden layer, and one output node.

The number of hidden layers and units in each layer is an important choice in a NN analysis, and are often determined experimentally through trial and error. One strategy to address this potential problem is to systematically try a range of architectures. Saccone et al. [24] evaluated 18 different architectures that included six different random number seeds and three different hidden layer sizes. Zandi et al. [25] evaluated 15 different architectures including 5 initial seeds and 3 hidden layer node sizes. Li et al. [19] and Pociot et al. [23] also evaluated several architectures with different numbers of hidden nodes.

While NN have been successful for pattern recognition in many fields, their success in linkage studies is debatable. Several linkage studies reported results demonstrating that the NN approach was able to detect at least one of the functional loci [4,18-24]. Many of these studies also reported false positive loci [4,18,19,22].

Each study describing NN applications for linkage studies indicate that further research is needed in this field. First, more work is needed to determine the best approach for selecting the most important loci in the NN [4,18,19,24]. Several studies [4,18,19,24] used a statistic called the contribution value (CV). The CV is calculated on each input node and is a function of the allele's contribution to the output node [4]. The CV's are usually rank ordered and the top markers that deviate most from the mean are selected as the most important for the NN [4,18-21,24]. More research is needed to determine how to use the CV most appropriately. For example, most previous applications selected the top 10 markers. A more appropriate alternative may be to select the top X% rather than top 10 as the most important. Another possibility is to derive an empirical distribution of CV's through permutation testing to use for selection of the most relevant loci [4]. Another more recent study used a sliding window approach to evaluate the classification accuracy of genetic regions and combinations of genetic regions [23]. In addition to selecting the most relevant loci, a method for placing statistical significance on loci is acutely needed [18,20,24].

In addition to selecting the most important loci and determining statistical significance, several other areas of uncertainty remain. For example, the interpretation of the weights needs to be investigated [20]. Further research is needed to determine the best way to use cross validation with NN for linkage analysis to prevent over-fitting data [19,24]. Finally, selecting the best NN architecture needs further investigation [20]. New approaches are discussed later in this review that address the problems related to variable selection, cross validation, and NN architecture selection.

### Neural Networks for association analysis

In addition to linkage studies, NN can be used for association studies. The same issues with data encoding and NN architecture exist for association analysis as well. As mentioned earlier, in association analysis, the data collected consist of genotypes for multiple markers in a sample of either case-control data or cases with family-based controls. The data can be encoded using three genotype levels (such as 0,1,2) [26] or as dummy variables that encode for the additive allelic effect as well as a nonlinear effect [27,28].

In contrast to linkage analysis, the number of publications using NN for association studies is slightly larger, and more real data applications have been performed [26,29-38]. Simulation studies have demonstrated the potential utility of NN, but also highlight the impact of architecture selection. In Curtis et al. [29] and Curtis [26], the NN architecture consisted of four input nodes that represent four markers coded as genotypes 0,1,2. There were two hidden layers, each with three nodes, and one output node, which had a target output of 0 for controls and 1 for cases. The results of Cutis et al. [29] study showed a lot of variability depending on the conditions simulated. Some data sets had highly significant single-locus results using the chi-squared test for association and no evidence for association using the NN, and vice versa. Through simulation studies, they show that the inclusion of NN analysis did not result in any significant loss in power and often produced an increase in power.

In their conclusion, Curtis et al [29] suggest that NN for association analysis can be developed in many ways. First, the NN architecture can be modified to optimize performance, including the number of inputs, number of hidden layers, and number of nodes in the hidden layer. Second, quantitative traits can be analyzed with NN by using the trait value as the target output. Finally, NN can provide a simple and practical method for dealing with multi-locus genotypes in case-control studies, and can be used to complement any traditional single locus analysis. They conclude that the NN approach is worthy of further exploration.

Their work is continued in North et al [35]. In this study, they examined the impact of adjusting many of the parameters involved in a NN analysis, including the number of training epochs, the training rate, and the architecture. They found that the performance of their NN algorithm was dependent on adjustments in all three parameters, particularly on the architecture. They simulated several different genetic models, demonstrating different modes of inheritance and found that the success of the NN analysis depended on the architecture chosen. Additionally, they found that the success of a particular architecture varied according to the genetic model simulated. For real data, when the underlying genetic model is unknown, their results underscore the challenge of finding the appropriate NN architecture for each individual dataset. Their work also highlights the potential of NN for applications to real data, however. They applied their NN algorithm to a real diabetes dataset and found that their NN approach had higher power than single locus tests due to the ability to consider multiple markers at one time, while only hypothesis testing the best model with permutation testing. This avoided the necessity of correcting for multiple testing. This study was extended in Curtis [26] to compare the power of the NN approach to other multimarker methods, including heterogeneity tests, logistic regression, and the UNPHASED algorithm [39]. The results of the extended simulation study empirically demonstrate the increased in power of the NN approach, even compared to other methods that also consider many markers simultaneously.

Where Curtis et al [29] and North et al [35] explore the impact of changing NN architecture, Shoemaker et al [37] uses simulated data to explore the impact of different types of input variables. They used fully connected feed-forward NN architecture with one input layer, one hidden layer, and one output layer representing affection status. They simulated multiple data types – including SNP variables along with quantitative and qualitative environmental traits. They examined the performance of NNs with each data type separately, and analyzed together. They found that NN have better predictive performance when all data types were used. The ability of NN to process a variety of input variables is a distinct advantage over other computational techniques, and this study empirically demonstrates this [37].

Real data applications in association studies have been largely positive. Serretti and Smelaldi [36] successfully used a back-propagation optimized NN with one hidden layer with 7 nodes to detect univariate genetic predictors of fluvoxamine response in mood disorders. Lin et al [33], and Tomita et al [38], used NN to identified significant associations in drug efficacy of interferons, and allergic asthma respectively. Matchenko-Shimko and Dube [34] used a bootstrapped estimate of predictor variable significance with NN to detect genetic and clinical risk factors of coronary artery disease. While the NN analysis detected significant effects, simultaneous Support Vector Machine (SVM) analysis did have higher predictive accuracy [34]. Giachino et al [31] also used NN to identify significant predictors, in Crohn's disease, but again saw that other analytical methods had higher predictive accuracy. The limited number of architectures evaluated in the NN analyses may explain these mixed results. This explanation is strengthened by the work of Penco et al [40]. Penco et al [40] identified significant genetic risk factors of venous thrombosis, and found NN to have better performance than other analytical methods using several architecture optimization algorithms. Falk [30] applied a NN approach to the Framingham Heart Study data, but did not find any significant predictors. A NN strategy was applied to detect risk factors associated with cardiovascular disease (CD) to classify individuals into normal and high blood pressure groups. Using a variety of input variables and architecture parameters, they were able to develop a NN model that classifies well in training sets, but were unable to develop a model with substantial predictive ability. They propose two possible explanations for their negative findings. This result might be due to an inappropriate design of the NN architecture or the low dependence of blood pressure on CD risk factors [30,41].

### Caveats of Neural Networks in genetic epidemiology

Thus far, we have reviewed several studies in linkage and association studies utilizing NN. While the successes of each study illustrate the potential of NN for genetic epidemiology, each study also highlights the potential caveats of the approach. Many studies detected the functional marker loci in simulated data, but also identified several false positive loci. Nearly every paper reviewed here discussed that NN appear to be a good approach for gene mapping studies especially when the goal is to identify multiple susceptibility genes simultaneously. However, more studies are needed to answer important questions about this approach. The following questions were posed by several of the papers reviewed here. First, how can we select important loci when we use a large set as input to the NN? Second, what is the best measure of loci relevance? Third, how can we put statistical significance on this measure of input relevance? Finally, what NN architecture is optimal for gene mapping studies? In the remainder of this review we discuss NN architecture as a potential area for improvement in the use of NN in genetic epidemiology.

The selection of the appropriate loci from a large pool of potential predictors is a concern with any model building approach, including NN. As genotyping technology rapidly advances, and genome-wide association studies become increasingly important in the field, the importance of variable selection in model selection is highlighted. Previous NN applications in genetic epidemiology have only addressed the variable selection problem in relatively small-scale studies.

Training of NNs is an artful challenge in any context. General concerns about over-parameterization are important to consider with any application of NNs. Concerns with selection of the appropriate NN architecture have been previously discussed, and choosing appropriate starting weights is an additional consideration. Typically, starting weights are randomly chosen values near zero [42]. This insures that the models begins very close to linearly, and becomes increasingly nonlinear during the weight optimization process. Weights cannot begin exactly at zero when using traditional back-propagation types of optimization because the algorithm would never move forward. The quality of a final NN model can also be greatly influenced by the choice of scaling used for the inputs. This is due to the fact that the input scaling determines the effective scaling of the weights in the NN layers. The input scaling must be carefully considered in the context of genetic epidemiology. Over-fitting is also cause for concern when training a NN. Often a NN has too many weights and will over-classify the data, reducing the power of the model to generalize to new, unseen data. Several approaches have been taken to prevent this. Early stopping rules are one method that has been employed to prevent over-fitting. Another common approach is to utilize internal model validation methods, like cross-validation to try to achieve a generalizable NN model. One final concern for training a NN is the possibility of multiple local minima. Because of the complex nature of the types of classification problems analyzed by NN, there are potentially many local minima, as well as one global minimum. The traditional back-propagation type of weight optimization is highly susceptible to becoming "trapped" in a local minimum if considerations are not taken to avoid such problems.

The study by Marinov and Weeks [21] addresses an important point related to NN architecture. In this study, the authors attempted to duplicate the study by Lucek et al. [20] in as much detail as possible. They used the same feed forward NN with 277 input nodes, 17 hidden nodes in one hidden layer, and two output nodes. Their goal was to achieve the same results obtained by Lucek et al. [20] in their NN analysis. Despite their efforts, Marinov and Weeks [21] were unable to duplicate the results produced by Lucek et al. [20]. Consequently, Marinov and Weeks [21] did multiple NN runs and identified different loci on each subsequent analysis. Some of the loci had consistent CV across runs while other had a very high CV in one NN analysis, and a very low CV in the next. The lack of repeatability from one analysis to the next is most likely due to the fact that training a NN involves minimizing a mean-squared error function. If the problem at hand involves a complex fitness landscape, there may be different local minima. Thus we may get a different result on each run of a NN. This was the case in the Marinov and Weeks [21] study, and most likely the explanation for why they were unable to duplicate the results of Lucek et al. [20] on the same data.

One potential explanation of the results in the Marinov and Weeks [21] study is that the appropriate NN architecture may not have been used. This is a well-known problem in the NN literature, and defining the network architecture is a very important decision that can dramatically alter the results of the analysis. Unfortunately, an exhaustive search over the space of all possible network architectures is computationally infeasible, even for modest size networks [43]. For example, with 10 input nodes, 1 output node, and 12 hidden nodes in a fully connected network, there are 4.46 × 10^43 ^possible solutions. If these networks could be trained and tested in one microsecond of CPU time, to test all these networks would take 1.41 × 10^30 ^years [43]. So even for a modest size example, it is quickly seen that an exhaustive search of all possible networks is not feasible.

There are a variety of strategies utilized for selection of the network architecture, in particular the number of hidden layers and nodes in the hidden layer. For example, the nodes in the hidden layer can be defined by N_H _= 2(N_P_N_S_)/(N_I _+ N_S_), where N_H _is the number of hidden units, N_P _is the number of observations, N_S _is the number of output nodes, and N_I _is the number of input nodes [44]. Another possibility is the cascade correlation learning architecture. Here the algorithm begins with a minimal network, then automatically trains and adds new hidden nodes one by one, creating a multi-layer network. However, once new hidden nodes are added to the network, its input-side weights are frozen. This approach has been advantageous since the NN learns quickly and determines its own size and topology [45]. Many of these approaches use a prediction error fitness measure, such that they select an architecture based on its generalization to new observations [46], while others use a classification error, or training error [43]. These methods are used to attempt to get the most learning out of the network, while trying to avoid over-fitting the data [43,45].

While these approaches seem reasonable, there are an effectively infinite number of architecture variations that can be selected. In addition, when the underlying model of the data varies from one data set to the next, an additional optimization procedure must be run on each data set to find the most appropriate architecture for each type of data. Therefore, we need to come up with new ways to select NN architecture to avoid the trial and error approach that has been previously employed.

## Optimization of Neural Network architecture

One potential solution to the architecture selection problem in NN is to evolve the NN architecture for each data set analyzed using an evolutionary computation approach. This will allow the user to avoid common pitfalls associated with having the wrong network architecture. Several evolutionary computation approaches have been proposed in the literature, including genetic algorithms [47], genetic algorithms in combination with back propagation [48], simulated annealing [49], and genetic programming [50]. The success of an artificial organism evolutionary approach to evolve NN has been successfully used in genetic epidemiology [40]. An extensive review of machine learning applications in the context of NN is found in Yao [51].

### Genetic Programming Neural Networks

We have developed a genetic programming optimized NN for association analysis [52], inspired by the previous work by Koza and Rice [50]. Genetic programming (GP) is a machine learning methodology that evolves computer programs to solve problems using Darwin's principle of "survival of the fittest" and evolution by natural selection. The GP evolves simple mathematical expressions as solutions to a problem [53]. The GP is very effective in searching highly nonlinear, multidimensional search spaces [50], such as those anticipated in complex diseases [54]. This ability to search complex fitness landscapes in parallel makes GP an attractive tool for optimizing NN to solve genetic epidemiology problems.

Genetic programming begins with an initial population of randomly generated computer programs, all of which are possible solutions to a given problem. This step is essentially a random search or sampling of the space of all possible solutions. Next, each of these computer programs are executed and assigned a fitness value that is proportional to its performance on the particular problem being solved. Then, the best computer programs, or solutions, are selected to undergo genetic operations based on Darwin's principle of survival of the fittest. Reproduction takes place with a subset of the best solutions, such that these solutions are directly copied into the next generation. Crossover, or recombination, takes place between another subset of solutions. This operation is used to create new computer programs by combining components of two parent programs. Thus, the new population is comprised of a portion of solutions that were copied (reproduced) from the previous generation, and a portion of solutions that are the result of recombination (crossover) between solutions of the parent population. This new population replaces the old population and the process begins again by executing each program and assigning a fitness measure to each of them. This is repeated for a set number of generations or until some termination criterion is met. The goal is to find the best solution, which is likely to be the solution with the optimal fitness measure.

To use GP to evolve NN architecture, the GP is constrained in such a way that it uses standard GP operators but retains the typical structure of a feed-forward NN. A set of rules is defined prior to network evolution to ensure that each GP solution maintains a structure that represents a NN. The rules used for this GPNN implementation are consistent with those described by Koza and Rice [50]. The flexibility of the GPNN allows optimal network architectures to be generated that contain the appropriate inputs, connections, and weights for a given data set [52].

**Figure 2:**
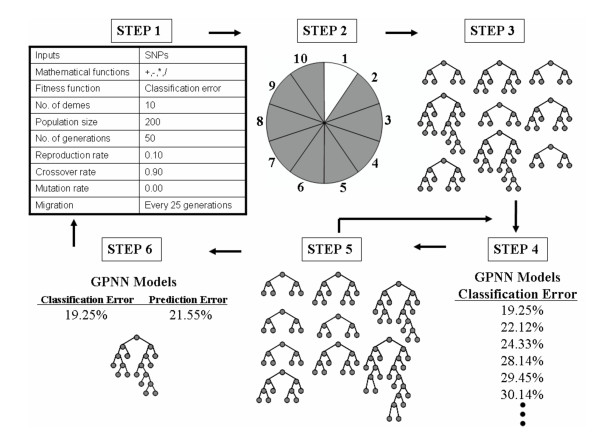
**Overview of the GPNN method (adapted from Ritchie et al. 2003)**. First, GPNN has a set of parameters to be initialized before beginning the evolution of NN models. Second, the data are divided into 10 equal parts for 10-fold cross-validation. Third, training begins by generating an initial population of random solutions. Fourth, each NN is evaluated on the training set and its fitness (classification error) recorded. Fifth, the best solutions are selected for crossover and reproduction using a fitness-proportionate selection technique. The new generation begins the cycle again. This continues until a stopping criterion (classification error of zero or limit on the number of generations) is met. At the end of the GPNN evolution, the overall best solution is selected as the optimal NN. Sixth, this best GPNN model is tested on the 1/10 of the data left out to estimate the prediction error of the model. Steps two through six are performed ten times with the same parameters settings, each time using a different 9/10 of the data for training and 1/10 of the data for testing. The loci that are consistently present in the GPNN models are selected as the functional loci and are used as input to a final GPNN evolutionary process to estimate the classification and prediction error of the GPNN model.

**Figure 3:**
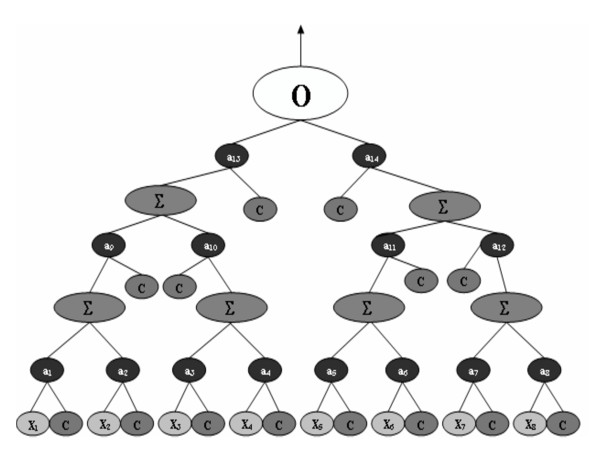
**A binary expression tree representation of a NN**. This is an example of one NN optimized by GPNN. The O is the output node, Σ indicates the activation function, a_i _indicates a weight, and X_1_-X_8 _are the NN inputs. The C nodes are constants.

An overview of the GPNN method is shown in Figure 2. Training the GPNN begins by generating an initial random population of solutions. Each solution is a binary expression tree representation of a NN, similar to that shown in Figure 3. The GP then evaluates each NN. The best solutions are selected for crossover and reproduction using a fitness-proportionate selection technique. A predefined proportion of the best solutions will be directly copied (reproduced) into the new generation. Another proportion of the solutions will be used for crossover with other best solutions. Crossover must take place such that the rules of network construction still apply. Next, the new generation, which is equal in size to the original population, begins the cycle again. This continues until some criterion is met at which point the GPNN stops. This criterion is either a classification error of zero or the maximum number of generations has been reached. In addition, a "best-so-far" solution is chosen after each generation. At the end of the GP run, the one "best-so-far" solution is used as the solution to the problem.

GPNN is implemented using 10-fold cross validation. Here, the data are divided into 10 equal parts. The GPNN evolves NN architecture using 9/10 of the data, and then tests this NN model on the 1/10 of the data left out. This is done 10 times, each time using a different 1/10 of the data for testing. The loci that are consistently present in the GPNN models are selected as the functional loci and are used as input to a final GPNN evolutionary process to estimate the classification and prediction error of the GPNN model.

While GPNN is effective in searching highly nonlinear, multidimensional search spaces, it is still susceptible to stalling on local minima [50]. To address this problem, GPNN can be run in parallel on several different processors. Several isolated populations, or demes, are created and a periodic exchange of best models takes place between the populations. This is often referred to as an "island model" [48]. This works by taking the "best-so-far" model from each processor and periodically copying it to all the other processors. So with n processors, at each exchange, any given processor will receive n-1 new models, replacing the n-1 worst models from that population. This exchange increases diversity among the models in the different populations. Following the set number of generations, the "best-so-far" models from each of the n processors are compared and a single best model is selected. Presumably, this model has the minimum error of all models generated [53].

In summary, GPNN optimizes the inputs from a pool of variables, the weights, and the connectivity of the network, including the number of hidden layers and the number of nodes in the hidden layer. Thus, the algorithm attempts to evolve appropriate network architecture for a given data set. GPNN also eliminates the need for the contribution value statistic, as GPNN selects the functional variables as input for the NN.

We have compared our GPNN to a traditional feed forward NN trained by back propagation using simulated data. The type of data simulations that we are most interested in consist of gene-gene interactions, where there are minimal main effects but strong interaction effects [55,56]. For these analyses, we simulated five gene-gene interaction models with two functional SNPs and eight non-functional SNPs. Each data set consisted of 200 cases and 200 unrelated controls [52].

We had two separate goals for our initial study comparing these two approaches. First, we wanted to determine if the GPNN was able to model gene-gene interactions as well as or better than a traditional NN. This was important to determine that the NN we evolved were functioning similar to a traditional NN. Since it is well known that NN can model nonlinear interactions, we wanted to validate that the GPNN performed as well at this task. For this question, the input to the NN included only the two functional loci. We used a dummy variable encoding for the genotypes where n-1 dummy variables are used for n levels [27]. Thus, for two functional SNPs, each with three genotype levels, there were a total of four NN inputs.

Second, we wanted to determine whether the GPNN was able to perform simultaneous variable selection and modeling of gene-gene interactions, as well as or better than a traditional NN [52]. In this stage of analysis, we used the functional loci as well as a subset of non-functional loci as input to the NN to determine if the GPNN could include the correct loci in the NN and model the interaction [52].

Using simulated data, we demonstrated that GPNN was able to model nonlinear interactions as well as a traditional NN. When given the functional SNPs, one would expect the NN to accurately model the data. We have shown that GPNN is also capable of accurately modeling the data. This demonstrates that GPNN is able to optimize the NN architecture such that the NN evolved is able to model data as well as a traditional NN. In addition, GPNN had improved power and predictive ability compared to a NN when applied to data containing both functional and non-functional SNPs. These results provide evidence that GPNN is able to detect the functional SNPs and model the interactions for a set of epistasis models. In addition, these are the two criteria we specified for considering GPNN an improvement over a traditional NN [52].

Since empirical studies suggested that GPNN has excellent power for identifying gene-gene interactions, a comparison of GPNN with traditional statistical methods was our next step. The goal of the next study was to compare the power of GPNN, Classification and Regression Trees (CART) and stepwise logistic regression (SLR) for identifying gene-gene interactions using data simulated from a variety of gene-gene interaction models. Our goal was to determine if GPNN is more powerful than traditional methods in the field [57]. Using simulated data with a variety of interactive genetic models, we showed that GPNN has higher power to identify gene-gene and gene-environment interactions than SLR and CART [57].

Additionally, after demonstrating that GPNN out-performed both a traditional NN and more traditional statistical methods, we wanted to know if GPNN would outperform a simple GP program [58]. Here we tested the power of GPNN and GP on simulated datasets generated under twenty different simulated epistasis models. Our results demonstrated that GPNN is more powerful than GP alone, and results in fewer false positive findings. This is most likely due to the confined search space of the GPNN approach, in comparison to a free form GP [58].

Next, we wanted to evaluate the power of GPNN for identifying high-order gene-gene interactions. Previous studies involved a relative small range of models, and we wanted to extend our simulation studies. We were interested in two, three, four, and five locus gene-gene interaction models with varying allele frequencies and heritability, and a constant relatively small sample size. Using simulated data, we showed that GPNN has high power to identify gene-gene interactions in the two and three locus interaction models, with small genetic effects. However, GPNN has reduced power for models with a very small genetic effect (heritability less than 1%) in addition to all four or five locus interaction models [59].

We were also interested in applying GPNN to a real data analysis in Parkinson's disease. GPNN was able to replicate the detection of a gene-environment interaction that had previously been detected using an exhaustive method, Multifactor Dimensionality Reduction (MDR) [59].

Based on the results of these studies, we have concluded that the GPNN is able to evolve NN architecture and perform as well as or better than a traditional NN. GPNN is able to model gene-gene interactions as well as a traditional NN when only the functional loci are used as input to the NN. More importantly, when non-functional loci are included as input to the NN, GPNN had much higher power than the traditional NN in identifying the functional loci and modeling the gene-gene interactions [52]. We have also shown that GPNN has higher power than traditional statistical methods and a stand-alone GP [57,58]. Additionally, empirical studies have shown that GPNN has excellent power to detect gene-gene interactions in a wide range of simulated models [59]. Perhaps most importantly, GPNN has been used to analyze a real data set and replicated a previous finding [59]. The results of these studies show the potential for using GP to optimize NN architecture.

### Grammatical Evolution Neural Networks (GENN)

Currently, we are exploring the use of another type of machine learning method-grammatical evolution (GE) – to evolve the inputs, weights, and architecture of NNs [60]. Grammatical Evolution (GE) is a form of evolutionary computation that allows the generation of computer programs using populations composed of linear genomes that are translated by a grammar [61,62]. Each individual consists of a binary genome divided into codons. Mutation can occur on individual bits along the genome, but crossover traditionally occurs only between codons. These codons are translated according to the grammar into a resulting phenotype (in this case, a functional NN). The resulting individual/phenotype can be tested for fitness and evolutionary operators are applied to create subsequent generations. By using the grammar to map a NN, GE separates genotype from phenotype (in the evolutionary computation process). Evolutionary operators function at the level of the binary string (the genome), while the selective pressure operates at the level of the accuracy of the NN (the phenotype). This allows for greater genetic diversity within a population than offered by other evolutionary algorithms. Understanding that the terminology used in describing GE and GENN is field specific, and can be confusing, thorough description and a glossary of terms can be found in [63].

Like GPNN, GENN improves upon the trial-and-error process of choosing an optimal architecture for a pure feed-forward back propagation neural network [60,63]. GENN optimizes the inputs from a large pool of variables, the weights, and the connectivity of the network – including the number of hidden layers and the number of nodes in the hidden layer. Thus, the algorithm automatically generates optimal neural network architecture for a given data set. GENN has been shown to improve power over a traditional BPNN, a random search algorithm, and GPNN in larger datasets [60,63]. This improved power is thought to be due to increased flexibility – GENN is able to evolve NN architecture more efficiently and with less computational cycles than GPNN [63]. By using a grammar, substantial changes can be made to the way that NN are constructed through simple manipulations to the text file where the grammar is specified. No changes in source code are required and thus, there is no recompiling. This results in a decrease in development time and an increase in flexibility. Linux software of the GENN method is currently available from the authors upon request, or at [64]. Details of the software package can be found in [63].

We anticipate these machine learning NN approaches will be important pattern recognition methods in the search for complex disease susceptibility genes. It will be important to understand the performance of these methods in the context of many of the challenges facing genetic epidemiology. Currently, the effect of several types of error common to genetic studies is being evaluated in the context of GENN (including genotyping error, missing data, phenocopy, and locus heterogeneity). Additionally, the scalability of the method is being evaluated. As the field has approached genome-wide association scans, it has become crucial that methods detect associations in the presence of thousands of genetic variables.

## Conclusion

In this paper, we have reviewed traditional back-propagation NN and their previous applications in genetic epidemiology for linkage and association studies. We have limited our discussion to back-propagation NN because they are the type of NN most commonly used in genetic epidemiology. Thus we did not examine the strengths or weaknesses of Hopfield nets, Radial basis function nets, or Bayesian networks. Additionally, we did not examine NN applications to haplotype estimation, phenotype assessment, or genetic counseling. While NN have been useful in many other fields such as economics, computer science, and engineering, their application in genetic epidemiology is in the exploratory phase. There are many heuristics that are required to perform a NN analysis including encoding the data, selecting the number or inputs and outputs, and constructing the NN architecture. With all of these choices, the results from NN analyses in genetic epidemiology have shown that NN can be effective in identifying functional loci. However, NN also tend to identify false positives as well and the results may vary from one NN analysis to the next. In addition, there is no solid foundation for selecting the functional loci from the set of inputs used in the NN. In order to evaluate the usefulness of NN for genetic epidemiology, it will be important to develop a strategy for selecting functional loci and constructing NN architecture. The GPNN strategy of Ritchie at al. [52]and the GENN strategy of Motsinger et al. [60,63]begin to address these issues and suggest that NN may provide an important piece of the analytical framework for the identification of susceptibility genes in common, complex diseases.

## Declaration of Competing interests

The authors declare that they have no competing interests.

## Authors' contributions

MDR conceived the concept for and the original draft of the manuscript. AAM compiled the up-to-date list of neural network applications, and updated the review.
